# Dr. Richard Douglas

**Published:** 1908-04

**Authors:** 


Dr. Richard Douglas, of Nashville, died at his home February 19, 1908, after a long illness of nephritis, aged 47 years. He was a native of Nashville where he was born December 20, 1860. He graduated from the medical department, University of Nashville, in 1881, and in 1882 took a post-graduate course at Jefferson Medical College, Philadelphia. He then returned to Nashville, where he practised his profession until shortly before his death.

Dr. Douglas was called to the chair of gynecology and abdominal surgery in the medical departments of the University of Nashville and Vanderbilt University, which at that time were represented by a single faculty. In 1895, when a severance of the two schools occurred, he became professor of gynecology and abdominal surgery in the medical department of Vanderbilt University, which position he held until failing health caused his resignation. In the early part of his professional career, Dr. Douglas spent several months abroad in the prosecution of his studies and after visiting the continental hospitals went to London, where he became the student of Mr. Granville Bantock.

Dr. Douglas was a frequent contributor to medical literature. His treatise on "Surgical Diseases of the Abdomen," published in 1904, attracted the attention of the surgical world. It is a book of great merit and is a textbook in many of the schools.

He was prominently identified with a number of medical societies, both American and foreign, and was one of the five American delegates to the International Medical Congress at Madrid, in 1903.

